# Fibromyalgia Patients Had Normal Distraction Related Pain Inhibition but Cognitive Impairment Reflected in Caudate Nucleus and Hippocampus during the Stroop Color Word Test

**DOI:** 10.1371/journal.pone.0108637

**Published:** 2014-10-02

**Authors:** Sofia Martinsen, Pär Flodin, Jonathan Berrebi, Monika Löfgren, Indre Bileviciute-Ljungar, Martin Ingvar, Peter Fransson, Eva Kosek

**Affiliations:** 1 Osher Center for Integrative Medicine, Department of Clinical Neuroscience, Karolinska Institutet, Stockholm, Sweden; 2 Dept. of Clinical Sciences, Karolinska Institutet, Danderyd Hospital, Danderyd, Sweden; University of Granada, Spain

## Abstract

The mechanisms causing cognitive problems in chronic pain patients are not well understood. We used the Stroop color word task (SCWT) to investigate distraction-induced analgesia, cognitive performance, and cerebral activation patterns in 29 fibromyalgia (FM) patients (mean age 49.8 years, range 25–64 years) and 31 healthy controls (HC) (mean age 46.3 years, range 20–63 years). In the first study, SCWT was used to investigate distraction-induced analgesia in FM patients. Two versions of the task were applied, one with only congruent color-word images and one with incongruent images. Pressure pain thresholds were assessed using a pressure algometer before, during, and following SCWT. In the second study, reaction times (RTs) were assessed and functional magnetic resonance imaging (fMRI) was used to investigate cerebral activation patterns in FM patients and HC during the SCWT. An event-related task mixing incongruent and congruent images was used. In study one, we found reduced pressure pain sensitivity during SCWT in both groups alike and no statistically significant differences were seen between the incongruent and congruent conditions. The study two revealed longer RTs during the incongruent compared to the congruent condition in both groups. FM patients had longer RTs than HC in both conditions. Furthermore, we found a significant interaction between group and congruency; that is, the group differences in RTs were more pronounced during the incongruent condition. This was reflected in a reduced activation of the caudate nucleus, lingual gyrus, temporal areas, and the hippocampus in FM patients compared to HC. In conclusion, we found normal pain inhibition during SWTC in FM patients. The cognitive difficulties seen in FM patients, reflected in longer RTs, were related to reduced activation of the caudate nucleus and hippocampus during incongruent SCWT, which most likely affected the mechanisms of cognitive learning in FM patients.

## Introduction

Fibromyalgia (FM) is a chronic pain syndrome, however, many FM patients also suffer from disturbed sleep, fatigue, mood disorders and cognitive impairment. The cognitive dysfunctions reported by FM patients [1]–[3] are referred to as “fibrofog” and can be more disabling than the pain itself [2], [4]. In particular, FM patients are easily distracted with difficulties focusing and redirecting attention [5], and have been reported to perform more poorly on tests assessing attention/executive function such as the Stroop Color Word Test (SCWT). The poor performance of FM patients was not related to disturbances in mood or sleep [6]–[8], but has been reported to correlate with high pain intensity [6] and high pain sensitivity [8].

In accordance with this, pain intensity has been shown to influence cognitive task performance in chronic pain patients using a Stroop paradigm [9]. Recently, Glass et al. (2011), reported aberrant brain activation in FM patients during a test targeting response inhibition. The authors hypothesized that overlapping networks are responsible for executive functioning tasks and pain processing, and that resources taken up by pain processing in FM patients would explain the inability to activate these networks during cognitive tasks [10].

Attentional resources are limited and therefore different stimuli compete with each other for attentional space. Based on this, a bi-directional interference between the perception of pain and the performance of cognitive tasks would be expected, i.e., not only would pain interfere with cognition but focusing on a cognitive task would also decrease pain perception. This is in line with previous findings showing that focusing on the pain can enhance pain perception and that pain perception can be decreased by distraction or cognitive tasks [11]–[13]. Modified versions of the Stroop interference test [14] have been used to assess cognitive modulations of pain perception and pain-related brain activity in healthy subjects. Incongruent Stroop was found to reduce heat pain sensitivity and increase activation of the cingulo-frontal cortex including the orbitofrontal and perigenual anterior cingulate cortex (ACC) as well as the periaqueductal gray (PAG) and the posterior thalamus in healthy controls [15]. Bantick et al. (2002) found that pain intensity scores for heat stimuli were significantly reduced when subjects took part in the cognitively more demanding task (incongruent stimuli) compared to the less demanding neutral task and this was accompanied by reduced activation in pain relevant brain areas such as insula, mid cingulate and thalamus. They also found that the peringual cingulate cortex and orbitofrontal cortex were more activated when painful stimuli and cognitive stimuli were presented together than what would be expected by a simple additive effect of the two [16].

However, using a more tonic pain stimulus it has been reported that the cognitive modulation of pain-related brain responses was not uniform but depended on behavioral strategy and that none of the brain areas showing attention-related responses, including dorsolateral prefrontal cortex (dlPFC) and posterior parietal cortex, were modulated by pain [13].

The authors concluded that pain stimuli in healthy subjects did not interfere with brain activities evoked by cognitively demanding tasks. Further, mild or moderate pain was not found to affect performance on a cognitive task, nor did the pain alter brain activity related to this task [17]. Therefore, the interference between pain and cognitive processing is complicated and state dependent.

To our knowledge, no previous studies have investigated the effects of chronic pain on attention-related cerebral responses during the SCWT, or the effects of Stroop on pain sensitivity in chronic pain patients. The aims of this study were two-fold. The first aim was to investigate the effect of cognitive load on distraction induced analgesia in FM patients and HC using the SCWT. In the SCWT the subjects are required to respond to the font color of color words, either congruent (“yellow” written in yellow) or incongruent (“yellow” written in green) [16]. Considering earlier findings of high cognitive load being related to increased distraction induced analgesia [16], we hypothesized that if FM patients have a normal ability to activate ACC during cognitive tasks, the higher cognitive load during the incongruent condition would cause a larger reduction of pain sensitivity compared to the congruent condition and no group differences in distraction-induced analgesia would be found between FM patients and healthy controls. If however, FM patients have a general inability to activate ACC, in line with their reduced ability to activate this structure during painful stimuli [18], then we would expect to find reduced distraction-induced analgesia in FM patients compared to healthy controls and no difference between the incongruent and congruent conditions.

Secondly, we wanted to use SCWT to investigate the effects of chronic ongoing pain on cerebral correlates of cognition in FM patients. As mentioned earlier, structures in the brain that are involved in cognition and pain perception are highly overlapping so mapping the neural correlates of this interference test would provide valuable knowledge as to which cerebral structures are impaired during cognition in FM. We chose the SCWT since it is a purely cognitive task known to activate the dorsal ACC (dACC) [19]. In healthy individuals, reciprocity has been found between activation of the dACC and dlPFC; individuals with high SCWT inference (longer reaction times (RT) during incongruent trials) exhibited higher task related dACC and lower dlPFC activation and vice versa [19]. Providing that FM patients do not have a generalized dysfunction of ACC, then an increased SCWT inference, reflected as higher task related dACC and lower dlPFC activation compared to HC would be expected. However, if FM patients have a more generalized dysfunction of ACC, either due to primary pathology or as a consequence of processing chronic pain [18], [20], [21], a reduced activation of dACC and no reciprocity between dACC and dlPFC would be expected during SCWT.

## Methods

### Subjects

Subjects were recruited by newspaper advertisement to participate in a multi-center experimental study (ClinicalTrials.gov identification number: NCT01226784) where FM patients were randomized to physical exercise or relaxation therapy. The current study was performed in the Stockholm cohort only and relies on baseline data before start of treatment, therefore no outcome of the clinical trial is reported in this manuscript.

FM patients: 31 women with FM were initially included in the study. However 2 patients had to be excluded (one due to not meeting MRI safety criteria and one due to inability to participate). The final cohort consisted of 29 FM patients average age 49.8 years (range 25–64 years). In study one, one participant was excluded due to inability to participate, leaving 28 participants. Four participants were unwilling or unable to participate in the fMRI part of the study; one participant reported falling asleep during the scan and one participant was not scanned due to technical failure of the equipment, leaving 23 participants.

Inclusion criteria for women with FM were to be of working age, 20–65 years, and meeting the ACR-1990 classification criteria for FM [22]. Exclusion criteria were high blood pressure (>160/90 mmHg), osteoarthritis in hip or knee, other severe somatic or psychiatric disorders, other primary causes of pain than FM, high consumption of alcohol (Audit >6), participation in a rehabilitation program within the past year, regular resistance exercise training or relaxation exercise training twice a week or more, inability to understand or speak Swedish, and not being able to refrain from analgesics, NSAID or hypnotics for 48 hours prior to examinations. One patient was on anticonvulsants and eleven were taking antidepressants (4 tricyclic antidepressants, 4 selective serotonin re-uptake inhibitors and 3 serotonin-noradrenalin re-uptake inhibitors). The daily intake of other drugs was as follows: NSAIDs 2 patients, acetaminophen 4 patients and tramadol 1 patient (no other opioid containing drugs were used on daily basis). All patients declared that they had refrained from hypnotics, NSAIDs, acetaminophen and tramadol/other analgesics at least 48 hours prior to study participation (48 hours before study one and 72 hours before study two (fMRI)). All patients had a physical exam by a specialist in rehabilitation medicine and filled in questionnaires regarding the impact of fibromyalgia (fibromyalgia impact questionnaire (FIQ) [23], hospital anxiety and depression scale (HADS) [24] and health related quality of life (Short Form - 36 (SF-36)) [25].

Healthy controls: 32 healthy female controls were recruited, one had to be excluded due to MR scan showing signs of neuroinflammatory changes, resulting in the final cohort consisting of 31 women, average age 46.3 years (range 20–63 years). All healthy subjects were interviewed regarding their health and completed relevant questionnaires. In study two one participant was unwilling to participate in the fMRI part of the study, one participant was not able to use the response box and one participant’s data was missing due to technical failure, this leaving 28 participants in study 2.

The subject characteristics are presented in table 1.

**Table 1:** Descriptive data for 29 FM patients and 31 HC, average values and range are presented.

	FM	HC	Group differences
**Age (years)**	49.8 (25–64)	46.3 (20–63)	NS
**FM dur (years)**	8.9 (0.5–19)	NA	NA
**Pain VAS (mm)**	45.3 (5–92)	0.6 (0–10)	P<0.0001
**FIQ**	63.1 (42.5–85.0)	6.8 (0–22.8)	P<0.0001
**HADS-D**	7.3 (3.0–16.3)	2.1 (0–7.0)	P<0.0001
**HADS-A**	8.8 (0–18)	3.2 (0–13.0)	P<0.0001
**SF-36 PCS**	30.2 (11.8–46.7)	55.2 (48.5–62.3)	P<0.0001
**SF-36 MCS**	36.5 (18.4–58.9)	50.6 (31.6–58.6)	P<0.0001

FM dur = duration of FM, Pain VAS = pain intensity rated on 100 mm visual analogue scale, FIQ = fibromyalgia impact questionnaire, HADS-D = Hospital anxiety and depression scale, depression ratings, HADS-A = Hospital anxiety and depression scale, anxiety ratings, SF-36 PCS = short form 36 physical compact score, SF-36 MCS = short form 36 mental compact score (original 0–100 scoring algorithms based on the summated ratings method). NA = not applicable.

The regional ethics committee in Stockholm approved the study and written informed consent was obtained from all participants.

### Material

#### Pressure algometry

Pressure pain thresholds (PPTs) were assessed using a pressure algometer (Somedic Sales AB). The pressure algometer had a gun shaped handle with a 1 cm^2^, circular, flat rubber tip on the end. It had a display that informed the experimenter how forcefully they were pressing and at which rate. The chosen rate was approximately 50 kPa/s. A response button was attached to the algometer and the subjects were instructed to push the button at the instant the pressure become painful. When this button was pressed, the current pressure froze on the screen. The algometer was calibrated for accuracy before each participant.

#### Stroop Color Word Test (SCWT)

Images were presented on a 17″ LCD screen with a resolution of 1024×768 pixels. Participants were given a response button box (Current designs) with four response buttons in the colors red, green, yellow and blue. The colors red, green and yellow together with the Swedish translation of these words made up the stimuli.

#### Functional magnetic resonance imaging

FMRI data was collected using a 3.0 tesla scanner (Discovery MR750, GE) and a 32-channel head-coil (MR instruments Inc). Foam wedges and headphones were used to minimize head motion and reduce perceived scanner noise.

### Procedure

Subjects were familiarized with the SCWT and assessed regarding pressure pain modulation during SCWT. They returned on the following day for the SCWT fMRI scan. The patients had to refrain from analgesics, NSAIDS and hypnotics 48 hours before the assessment of pain modulation during SCWT and 72 hours before the fMRI scan. Prior to fMRI a 3-plane localizer was performed followed by ASSET-calibration for parallel imaging.

#### Pressure pain modulation during SCWT

Two paradigms were used in the SCWT test, one paradigm using only classic Stroop images: color words written in incongruent colors (for instance the word “green” in red color), and one control task with color words written in congruent colors (the word “red” in red color). Both paradigms lasted 10 minutes, each stimulus was presented on the screen for 2 seconds and the interstimulus interval (ISI) was three seconds. During the ISI a fixation cross appeared after a second and stayed on the screen for one second. The experimenter orally instructed the participants to respond based on the color of the text and not the word that they were reading. Participants were explicitly instructed to only use the red, green and yellow buttons as blue stimuli was not used. A test run was performed using congruent images and the experimenter ended the run when confident that the participant had familiarized themselves with the speed of the experiment and the location of the buttons. Participants pressed the response buttons with the index, middle and ring finger on the right hand.

To acquaint the participants with the algometer it was demonstrated at the upper arm before testing at the thigh. PPTs were assessed eight times during the congruent and incongruent condition respectively. Twice before the beginning of the paradigm, then at 60, 180, 300, 420 and 540 seconds into the task, and finally once 10 minutes after the task had ended. Blood pressure and heart rate was measured using an automated blood pressure monitor once before the beginning of the task, then 90, 210, 330 and 450 seconds into the task, and finally 10 minutes following task conclusion. The last measurement taken 10 minutes following the conclusion of the first task was also used as the initial measurement prior to the second task.

Half of the FM patients as well as healthy controls started with the incongruent condition followed by the congruent condition, and vice versa. In order to avoid sensitization, PPTs were initially assessed at the right thigh following the left thigh in half of the participants and vice versa in a counterbalanced order.

#### SCWT during fMRI

**Figure 1:**
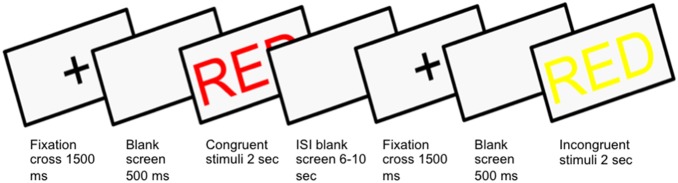
Example of one congruent and incongruent trial showing the fMRI set-up. Stimuli were presented in Swedish, but the English translation is used here for illustrative purposes.

Imaging parameters were as following: single shot EPI with a flip angle of 90°, TE 30 and TR 2500. Field of view (FOV) 28.8, 48 axial slices with a slice thickness of 3 mm, no slice spacing, which were acquired interleaved. There were two runs lasting 10 minutes. There were 84 images in each run with an even distribution of congruent and incongruent trials; the order of presentation of congruent and incongruent images was randomized. Interstimulus asynchrony was jittered between six and ten seconds, averaging at eight seconds. The stimulus was presented on a screen (24×32 cm) behind the participants, which was made visible via a mirror placed on the coil. A fixation-cross preceded each stimulus 500 ms followed by a blank screen for 1500 ms. The stimulus was on for 3000 ms. The word “Ready” appeared on the screen prior to the first fMRI run, the experimenter asked the participant to read the word on the screen out loud to ensure that they were positioned in such a way that they could see the words on the screen, as well as to confirm their ability to read the words used as stimuli. The paradigm was run using Neurobehavioural Systems Presentation software and is illustrated in figure 1. T1 weighted structural images were acquired in coronal orientation for anatomical reference purposes and T2 weighted anatomical images for screening for structural anomalies.

### Statistics

#### Analysis of pressure pain modulation during SCWT

In order to assess the effect of SCWT on pressure pain sensitivity the relative change in PPTs at m. quadriceps during SCWT was analyzed. In analogy with our previous studies of pain inhibitory mechanisms the PPTs were normalized (i.e., each PPT value was divided by the individual’s first PPT measure) [26]. The effect of SCWT on PPT was analyzed using a repeated measures ANOVA with the within-subject factors CONGRUENCY (2 levels, congruent and incongruent SCWT) and TIME (7 levels, before, 5 times during and 10 minutes following SCWT) and the between-subject factor GROUP (FM patients and controls). The effect of SCWT on blood pressure and heart rate was analyzed by repeated measures ANOVA with the within-subject factors CONGRUENCY (2 levels, congruent and incongruent SCWT) and TIME (6 levels, before, 4 times during and 10 minutes following SCWT) and the between-subject factor GROUP (FM patients and controls). The difference in reaction time was assessed by repeated measures ANOVA with the within-subject factors CONGRUENCY (2 levels, congruent and incongruent SCWT) and the between-subject factor GROUP (FM patients and controls).

Greenhouse-Geisser corrections were used in case of significant test of sphericity.

Group differences in absolute PPTs at baseline and reaction times were assessed by Students’ independent t-test. Post hoc analysis of intraindividual differences in PPTs over time and reaction times were assessed by Students’ paired t-test. Group differences in the number of errors during the SCWT, were analyzed by Independent Samples Mann-Whitney U-test.

#### Analyses of fMRI-data

Data was analyzed using SPM8 (Wellcome Trust Centre for Neuroimaging, University College London, UK). EPI images were realigned and then normalized to the canonical EPI-template in standard Montreal Neurological Insititute (MNI) space. Finally the images were smoothed using an 8 mm full-width-at-half-maximum (FWHM)-kernel. For each individual subject, first-level analysis was performed using a fixed effects analysis compounding both scans into the same general linear model (GLM). Stimulus onset times of congruent and incongruent stimuli were entered as regressors of interest, and movement parameters were entered as covariates of no interest. All regressors were convolved with the canonical hemodynamic response function (peak positive BOLD response after approximately 5–6 seconds after stimulus onset, return to pre-simuli baseline after 10–12 seconds) as implemented in SPM8 before they were entered into the general first level linear model [27]. Brain activity at a group level was assessed using a random, second level analysis performed in SPM8 using the parameter files from the individual contrasts files that pertained to the regressors of interest at the first level. To evaluate within-group increases/decreases in brain activity, one-sample T-tests were used (activation maps thresholded at p<0.05, family-wise error corrected), whereas between-group differences in brain activity was carried out using 2-sample T-tests (activation maps thresholded at p<0.001 uncorrected). Clusters of activity smaller than 20 contiguous voxels were not reported. All T- and 2 sample T-tests at the group level were performed with the general linear model framework as implemented in SPM8.

## Results

### Subject characteristics

The subject characteristics are presented in table 1. As expected FM patients had higher ratings of depression and anxiety as well as reduced health related quality of life compared to HC.

### Pressure pain modulation during SCWT

**Figure 2:**
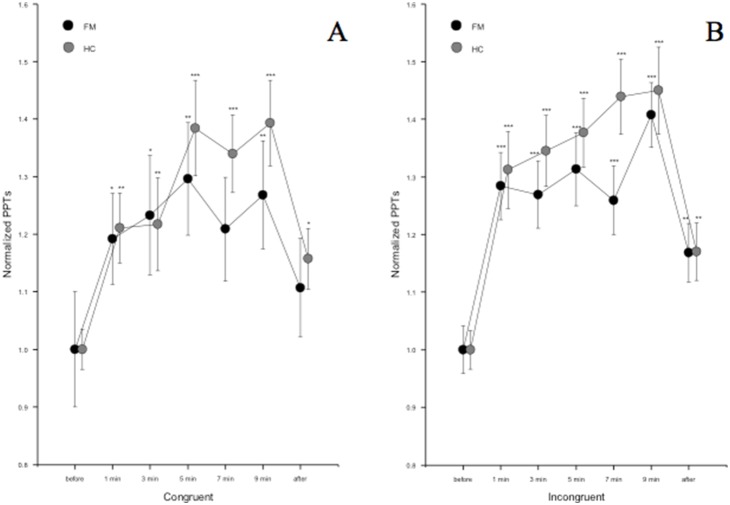
Average normalized PPTs ± SEM during a) congruent and b) incongruent SCWT in FM patients and HC. PPTs increased in both groups alike during congruent as well as incongruent SCWT. There were no statistically significant differences between congruent and incongruent SCWT regarding the modulation of PPTs. * = p<0.05, ** = p<0.01, *** = p<0.001. The normalization was performed as follows: each PPT value was divided with the individual’s first PPT at baseline and the curves were adjusted (by adding a coefficient) so that the baseline value always corresponded to 1.

The average absolute PPT at m. quadriceps was 161 kPa in FM patients and 292 kPa in healthy controls (p<0.0001) at baseline and the absolute PPT was lower in FM patients compared to controls at all times during incongruent as well as congruent SCWT (p<0.0001). The effect of SCWT on normalized PPTs is shown in figure 2. There was a statistically significant effect of TIME (d = 4.69, F = 35.28, p<0.0001), but no statistically significant effect of CONGRUENCY or GROUP and no statistically significant interactions were seen. The normalized PPTs were higher at all times during incongruent SCWT compared to baseline in both groups (p<0.0001) and also at 10 minutes following the SCWT in both groups (p = 0.001).

PPTs were significantly higher in FM patients during congruent SCWT at all times compared to baseline (p<0.05) with the exception at 7 min, but no statistically significant difference was seen 10 min following SCWT compared to baseline. In HC PPTs were higher at all times during congruent SCWT compared to baseline (p<0.01) and remained elevated 10 min following Stroop (p = 0.016).

### Blood pressure (BP) and heart rate (HR) during SCWT

There were no statistically significant group differences in BP or HR at baseline. Regarding systolic BP, there was a statistically significant effect of TIME (d = 3.20, F = 5.40, p = 0.001), CONGRUENCY (d = 1, F = 9.57, p = 0.003) but not GROUP and no statistically significant interactions were seen. During incongruent SCWT systolic BP increased at 90 s compared to baseline (from 117 to 120 mmHg, p = 0.002) and then returned to baseline. No statistically significant differences from baseline were seen during congruent SCWT.

Regarding diastolic BP there was a statistically significant effect of TIME (d = 2.43, F = 7.03, p<0.0001), but not CONGRUENCY or GROUP and no statistically significant interactions were seen. During incongruent SCWT diastolic blood pressure increased at 90 s (84 mmHg, p<0.0001), 210 s (83 mm Hg, p = 0.008) and 330 s (83 mmHg, p = 0.013) and then returned to baseline values (81 mm Hg), whereas it increased at 90 s (from 81 to 83 mmHg, p = 0.003) during congruent SCWT and then returned to baseline.

Regarding HR there was a statistically significant effect of TIME (d = 2.34, F = 3.92, p = 0.017) but not CONGRUENCY or GROUP and no statistically significant interactions were seen. During incongruent SCWT there was a statistically significant HR increase from baseline (69 bmp) at 210 s (71 bmp, p = 0.027) and at 330 s (71 pbm, p = 0.022), no other significant increases from baseline were seen during incongruent or congruent SCWT.

### Stroop prestanda

**Figure 3:**
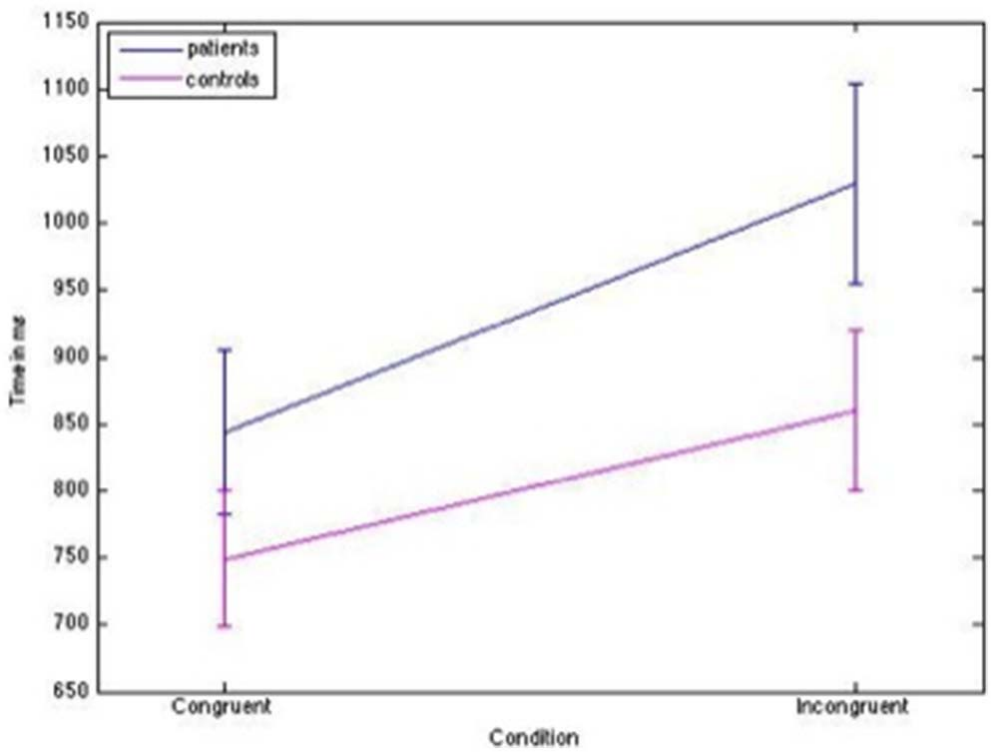
Average reaction times (s) ± SEM during congruent and incongruent SCWT. FM patients had longer reaction times than controls in both conditions (congruent p = 0.027, incongr p = 0.005), but the difference was more pronounced during incongruent SCWT (p = 0.023).

Average RTs for HC were 870.57 ms (standard deviation (SD)  = 165.42 ms) for incongruent stimuli, and 758.20 ms (SD = 121.47 ms) for congruent stimuli. The average RTs for FM patients were 1017.81 ms, (SD = 207.10 ms) for incongruent stimuli and 836.53 ms (SD = 149.25 ms) for congruent stimuli (Fig. 3). There was a statistically significant effect of CONGRUENCY (d = 1, F = 106.87, p<0.0001) and GROUP (d = 1, F = 7.61, p = 0.008) and a significant CONGRUENCY×GROUP interaction (d = 1, F = 5.49, p = 0.023).

Compared to HC, FM patients had longer RTs during incongruent (p = 0.005) as well as congruent (p = 0.027) SCWT, but the group difference was more pronounced during the incongruent than the congruent task. This is illustrated in figure 3.

There were no significant group differences regarding the number of errors made during congruent (HC hit ratio 99.99%, FM hit ratio 99.99%, p = 0.850) or incongruent (HC hit ratio 99.81%, FM hit ratio 99,76% p = 0.426) SCWT.

### fMRI results

#### Main effect of SCWT

**Figure 4:**
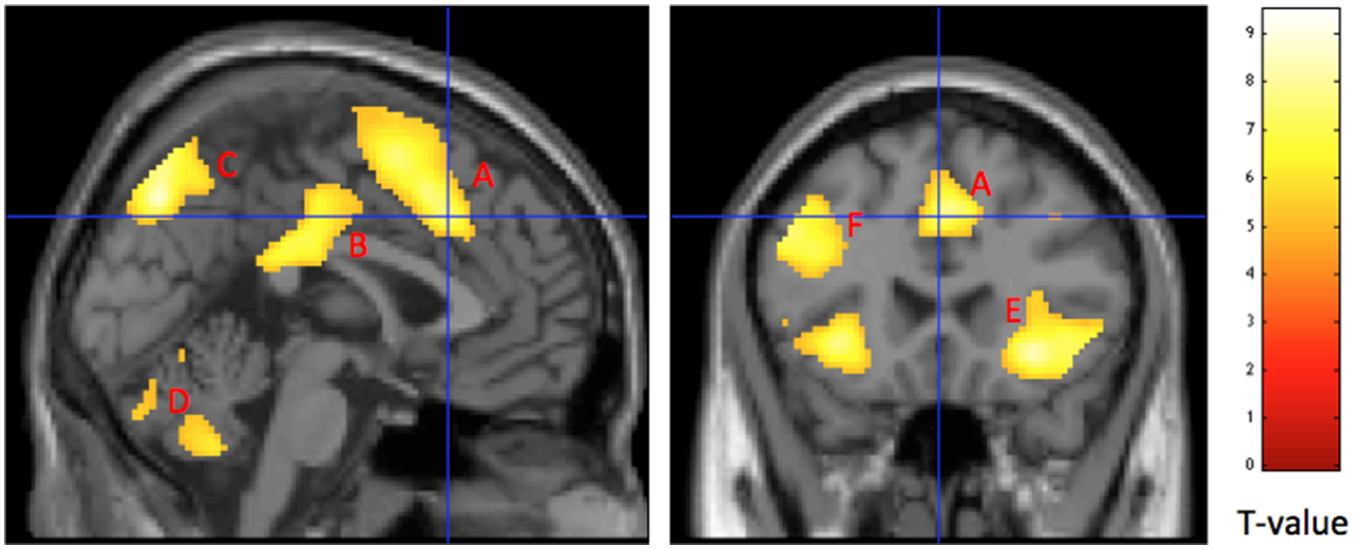
Main areas of interest representing cerebral activation when contrasting incongruent >congruent stimuli for FM patients (n = 23) and HC (n = 28) together. Results are reported at p<0.05, FWE corrected for multiple comparisons. A: dorsal anterior cingulate cortex (dACC), B: posterior cingulate cortex (PCC), C: precuneus, D: cerebellum, E: Insula (bilateral) and F: dorsolateral prefrontal cortex (dlPFC).

The main effect of the contrast incongruent – congruent when both groups were analyzed together revealed an expected activation network including the parietal lobe, dorsal ACC (dACC), posterior cingulate cortex (PCC), precuneus, insula and dlPFC, (Figure 4, Table 2).

**Table 2:** Representation of cerebral activation when contrasting incongruent >congruent stimuli for FM patients and HC together.

Anatomical Region	ClusterSize	X	Y	Z	PeakZ-value	P-value
Left Inferior Parietal Lobe	14730	−36	−52	46	7.29	p<0.0001
Cerebellum	3867	30	−52	−24	7.20	p<0.0001
dACC	2252	6	18	42	7.03	p<0.0001
Right Insula	1358	30	22	−8	6.92	p<0.0001
Left Fusiform Gyrus/Cerebellum	2573	−44	−52	−18	6.77	p<0.0001
Right Mid Frontal Lobe	847	38	−2	64	6.14	p<0.0001
Right Mid/PosteriorCingulate Cortex	716	−4	−20	32	6.00	p<0.0001
Right Superior Frontal Lobe	133	24	46	38	5.53	p = 0.001
Right Mid Frontal Lobe	210	40	38	34	5.07	p = 0.001
Left Thalamus	69	−8	−18	4	5.05	p = 0.004
Right Calcarine Sulcus	68	12	−70	8	4.94	p = 0.03

Results are reported at p<0.05, FWE corrected for multiple comparisons at cluster level.

#### Between group effects

**Figure 5:**
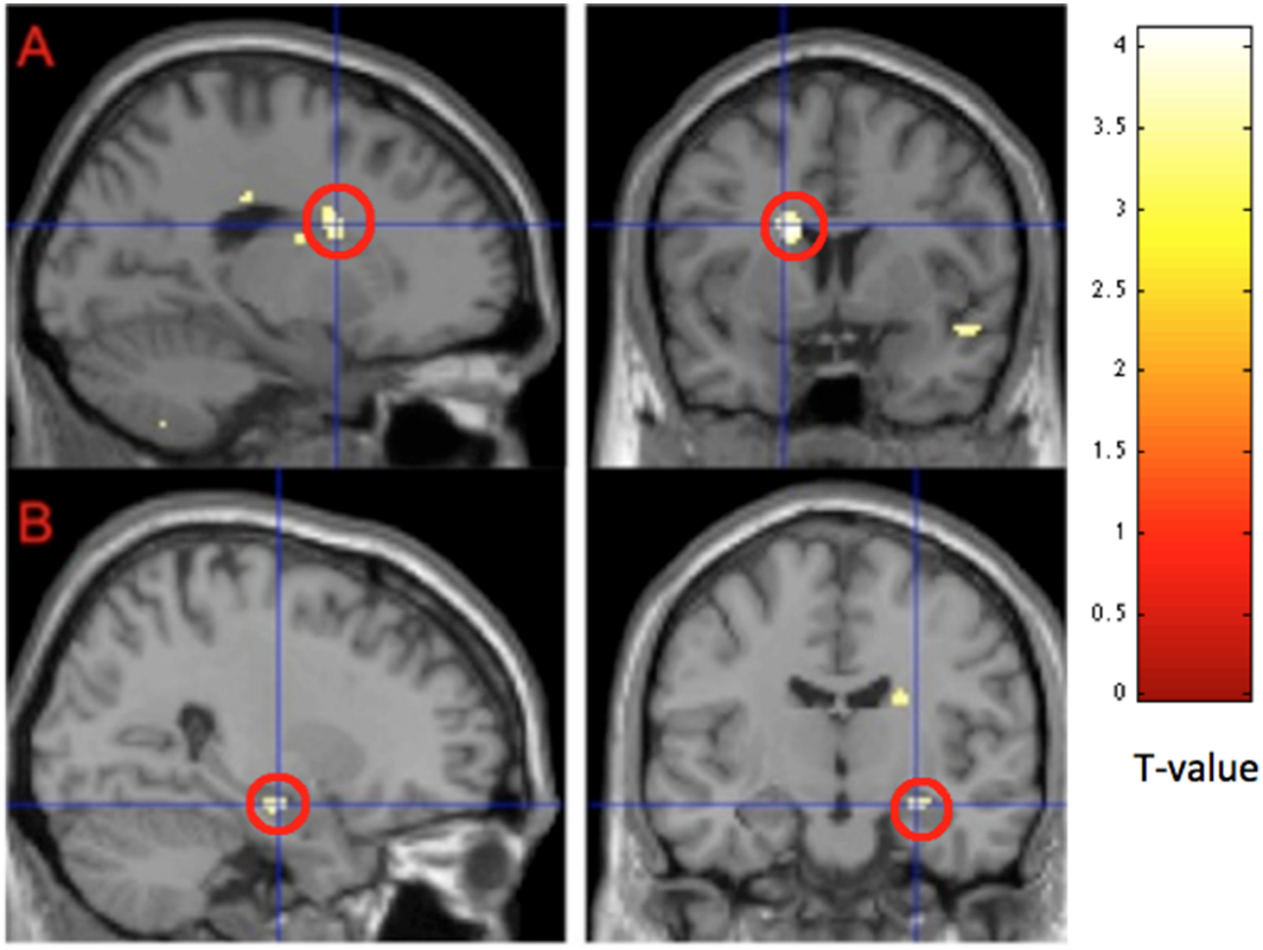
Representation of cerebral activation when contrasting HC>FM for incongruent >congruent stimuli. HC had significantly higher activation than FM patients in **A)** the caudate nucleus (p<0.0001, peak voxel) and **B)** the hippocampus (p<0.001, peak voxel). Results are reported a threshold setting of p<0.001, p values are at peak voxel, not corrected for multiple comparisons.

FM patients showed no regions with more activity than HC for incongruent >congruent images. When contrasting HC>FM we found greater activation in the caudate nucleus (bilaterally), temporal areas encompassing the hippocampus and the lingual gyrus (Figure 5, Table 3). The results remained also when FM patients on antidepressants and anticonvulsants were excluded from the analysis, but at a lower level of significance.

**Table 3:** Representation of cerebral activation when contrasting HC>FM for incongruent >congruent stimuli.

Anatomical Region	ClusterSize	X	Y	Z	PeakZ-value	P-value
Right Caudate Nucleus	76	22	−14	24	3.78	p<0.0001
Right Mid Temporal Lobe	154	42	−2	−16	3.73	p<0.0001
Right Hippocampus	26	26	−14	−16	3.52	p<0.0001
Right Superior Temporal Pole	32	44	−40	24	3.50	p<0.0001
Right Lingual Gyrus	37	6	−56	0	3.50	p<0.0001
Left Hippocampus	28	−38	−24	−8	3.50	p<0.0001
Left Caudate Nucleus	49	−14	6	22	3.49	p<0.0001

Results are reported at threshold setting of p<0.001, p values are at peak voxel, not corrected for multiple comparisons. Smallest reported cluster-size is 20 voxels.

## Discussion

We found a normal inhibition of pressure pain sensitivity in FM patients compared to healthy controls when engaging in the cognitive task. This was independent of the cognitive demand/load thus suggesting that distraction of attention away from painful stimuli, rather than engaging in the cognitive task per see was mediating the decreased pain sensitivity. To our knowledge, this is the first evidence that FM patients have a normal ability to regulate pain sensitivity during distraction. As expected, FM patients had slower RTs during the cognitive task, but did not make more mistakes. Several brain regions showed more profound activation in both groups alike during the cognitively more demanding (incongruent) task compared to the congruent task, mainly cerebellum, inferior parietal lobe, dACC, PCC and right insula. FM patients did not show increased task related activation of ACC. Instead reduced task related cerebral activations were seen when FM patients were compared to healthy controls in brain regions implicated in cognitive processing and memory such as the caudate nucleus, and hippocampus.

### Pain modulation during SCWT

We found the same relative reduction in pressure pain sensitivity in both groups when engaging in the SCWT task. Furthermore, the cognitive load did not influence pain sensitivity. To our knowledge, this is the first evidence of normal activation of top-down pain inhibitory mechanisms in FM patients and our results indicate that distraction from pain stimuli can reduce, although not normalize, pain sensitivity in FM patients.

The effect of distraction on heat pain sensitivity has been studied previously in healthy individuals, and it was found that the PAG is activated during distraction away from the painful stimuli. The authors also reported a correlation between the degree of PAG activation and reduced pain sensitivity [28]. These findings were later supported by a study showing that distraction from heat pain stimuli by SCWT reduced pain sensitivity and decreased cerebral pain related activations, particularly in the so called “medial pain system” [15]. However, compared to pain stimulation without distraction, distraction increased the pain related activation in OBFC, ACC, thalamus and PAG, and these activations were related to reduced pain sensitivity, indicating the role of these structures in top-down pain modulation [15]. In fact, PAG and rostral ACC (rACC) has been shown to be implicated in opioid as well as placebo analgesia [11]. We have previously demonstrated that FM patients have an augmented processing of evoked pressure pain stimuli but impaired ability to activate cerebral sites related to descending pain inhibition, i.e., rACC and brainstem/PAG [18]. Furthermore, FM patients had reduced connectivity between rACC and the brain’s pain inhibitory network, including the brainstem/PAG, during evoked pressure pain [21]. Our findings are in accordance with behavioral studies showing an inability of FM patients to activate endogenous pain inhibitory mechanisms, such as conditioned pain modulation (CPM) [29], [30] and exercise induced analgesia (EIA) [26], [31], [32]. However, since CPM and EIA involves using a painful conditioning stimulus it has been proposed that deficient CPM and EIA in FM patients could be due to an ongoing activation of these pain inhibitory networks by the chronic pain, thus precluding further activation [18], [26] and/or by activation of pain facilitatory mechanisms by the painful conditioning stimuli [26]. That FM patients are able to modulate pain sensitivity during a cognitive task would support the latter. Moreover our data would indicate that the higher order cognitive areas, such as the OBFC are functioning normally in FM patients during SCWT thus enabling the patients to distract from pain stimulation and explaining the normal pain modulation seen during SCWT, however this requires further study.

The current findings are in accordance with a pilot study conducted in our group showing normal ability of FM patients to inhibit pressure pain sensitivity during a stressful variant of the incongruent SCWT accompanied by profound BP and HR increases (unpublished data). The paradigm of the present study was chosen to ensure that the increase in BP and HR would be very modest, in fact, it was only seen during the first part of the SCWT, offsetting baroreceptor analgesia as an likely explanation [33].

### Cerebral activation during SCWT

FM patients had longer RTs during SCWT compared to controls and the group difference was even more pronounced during the incongruent trials, however they did not make more mistakes. This is in accordance with previous studies [6]–[8], [34], [35] and indicates impaired regulation of attention/executive function in FM. However, it was not reflected in an increase of task related dACC activation as hypothesized based on the previous findings in healthy controls [19]. This evokes two new hypotheses regarding the dACC in FM; either that FM patients have a general dysfunction of the ACC, as they are not showing activation patterns as healthy poor performers would be expected to, or that other systems are failing. Further exploratory analysis of our findings provided support for the latter showing reduced activation in the caudate nucleus and hippocampus during SCWT. Malfunction of the caudate nucleus in FM has been suggested before based on findings of decreased cerebral blood flow [36], although this is not a uniform finding [37], [38]. Furthermore, in hippocampus, aberrations of metabolites as well as glutamate concentrations have been reported in FM [39], [40]. Although caution should be made when drawing parallels between study one and study two in the current article, the behavioural results indicate normal functioning of higher order cortical areas, which was supported by the fMRI data.

The ability to learn new action schemas is required in order to perform well on the SCWT. Caudate nucleus and hippocampus are areas that are connected to each other and involved in learning as well as pain modulation, making them highly clinically relevant in FM syndrome. Normal functioning of the caudate nucleus depends on dopamine (DA), and DA modulates reciprocally with serotonin [41]. Reduced concentrations of DA, noradrenaline (NA) and serotonin metabolites have been reported in the cerebrospinal fluid of FM patients [42], and furthermore, treatment with a DA increasing drug has shown promising results in FM [43]. Studies that have investigated pain and cognition in DA related disorders, such as attention deficit hyperactivity disorder (ADHD) and Parkinson’s disease, have found similarities with FM. Patients with Parkinson’s disease had more errors and longer RTs when doing the Stroop task [44], [45], and presented other cognitive problems such as difficulties shifting between action schemas, which has been suggested to be related to low DA levels in the caudate nucleus [46]. ADHD patients have been reported to experience not only difficulties with attention, but also problems with motor skills and they often report pain [46]. Although studies of pain in ADHD patients are scarce, one study reported widespread pain in 80% of the ADHD participants [47] and ADHD subjects had lower tolerance to evoked pain than HCs [48]. Following treatment with methylphenidate (Ritalin), a substance increasing extracellular DA and NA, pain tolerance was elevated, though not completely normalized, in ADHD patients [48]. A history of childhood ADHD has been reported to be more frequent in FM patients [49] and a larger proportion of FM patients scored high on an ADHD diagnostic scale compared to other chronic pain patients [50]. Therefore, it is possible that ADHD and FM share some common DA related pathology. Further support for this comes from PET studies showing reduced dopamine (D2) receptor binding potential in the caudate nucleus in FM [51] as well as in ADHD [52].

### Limitations

Contrary to our hypothesis and to previous findings [51], the distraction-induced analgesia was not influenced by cognitive load in our HC or FM patients. However, we cannot exclude the possibility that the difference in difficulty between the incongruent and congruent task in this study was insufficient. Therefore it is possible that a more difficult task would have further improved the distraction-induced analgesia in both groups.

We did not assess the cerebral correlates of pain modulation during SCWT with fMRI, which would have provided additional important information. Also, contrary to previous studies, we did not assess the effects of evoked painful stimuli on cognitive function. Combining these two approaches during fMRI in future studies could improve our understanding of the cerebral interactions during pain processing and cognition. Finally, we did not want to exclude patients on antidepressants/anticonvulsants since this would risk bias our FM cohort towards less affected individuals.

## Conclusions

To our knowledge, this is the first evidence that FM patients have a normal ability to regulate pain sensitivity when focusing on a cognitive task. We found evidence of slower cognitive processing in FM patients related to task difficulty. Contrary to our hypothesis, no evidence of ACC dysfunction during SCWT was seen. Instead FM patients had reduced activation of caudate nucleus and hippocampus during SCWT in line with problems learning new actions schemas.
